# Mental wellbeing amongst younger and older migrant workers in comparison to their urban counterparts in Guangzhou city, China: a cross-sectional study

**DOI:** 10.1186/1471-2458-14-1280

**Published:** 2014-12-16

**Authors:** Jie Li, Shu-Sen Chang, Paul S F Yip, Juan Li, Lucy P Jordan, Yunge Tang, Yuantao Hao, Xingmei Huang, Ning Yang, Chaoqi Chen, Qiaomei Zeng

**Affiliations:** Guangzhou Psychiatric Hospital, No 36 Mingxin Road, Liwan, Guangzhou, 510370 Guangdong China; The Hong Kong Jockey Club Centre for Suicide Research and Prevention, The University of Hong Kong, 5 Sassoon Road, Pokfulam, Hong Kong, SAR China; Department of Social Work and Social Administration, The University of Hong Kong, Pokfulam Road, Pokfulam, Hong Kong, SAR China; Ju Shan Hospital, No 910, Daguan Road, Dayuan Township, Taoyuan, 337 Taiwan; Family Planning Research Institute of Guangdong Province, Guangzhou, Guangdong China; School of Public Health, Sun Yat-Sen University, Guangzhou, Guangdong China

**Keywords:** China, Guangzhou, Migration, Mental health, Wellbeing, SF-36, WHO-5

## Abstract

**Background:**

There has been a dramatic increase in internal migrant workers in China over recent decades, and there is a recent concern of poor mental health particularly amongst younger or “new generation” migrants who were born in 1980 or later.

**Methods:**

A cross-sectional study was conducted in Guangzhou city between May and July in 2012. Mental wellbeing was measured using the World Health Organization Five-item Well-Being Index Scale and the 36 Item Short Form Health Survey mental health scale. Linear and logistic regression models were used to investigate the differences between migrant workers and their urban counterparts and between younger and older migrants.

**Results:**

Migrant workers (n = 914) showed a small but significant advantage in mental wellbeing compared to their urban counterparts (n = 814). There was some evidence for age modification effect (p for interaction = 0.055-0.095); better mental wellbeing in migrants than urbanites were mainly seen in the older compared to the younger group, and the difference attenuated somewhat after controlling for income satisfaction. Older migrants showed better mental health than younger migrants. Factors that were independently associated with poor mental health in migrants included being male, longer working hours, and income dissatisfaction, whilst older age, factory job, high income, and increased use of social support resources were associated with reduced risk.

**Conclusions:**

Efforts to promote mental health amongst migrant workers may be usefully targeted on younger migrants and include measures aimed to improve working conditions, strengthen the social support network, and address age-specific needs.

## Background

The last three decades have seen a dramatic rise in internal migration in China. In 2010 there were 261 million migrant workers, accounting for nearly one fifth of China’s population [1]. These migrants mostly come from rural areas in western and central China and move to cities in the eastern and southern coastal regions. They are not allowed to change their registered status from rural to urban under the household registration (*hukou)* system, which was established in the late 1950s to restrict rural-to-urban migration [2, 3]. These migrants are thus classified as temporary residents in host cities and are largely excluded from access to benefits such as subsidised housing, social security, and medical benefits available to registered urban residents [4–6]. They commonly take up low paid manual jobs and work long hours [6], mostly live in conditions that are very basic and limited [7, 8], and often experience stigma and discrimination [9, 10].

Previous studies of Chinese migrant workers’ mental health have been limited and produced various findings; some showed poorer mental health status in migrants than the urban counterparts [11, 12], whilst others showed the contrary pattern [13] or similar levels of psychological distress in the two groups [14]. Compared to mental health, the physical health of Chinese migrants has received more attention and has been better studied. For example, there have been a number of studies on infectious diseases, maternal heath, and occupational disease and injuries in this population [15]. Overall, previous studies showed some evidence for better physical health status in migrants than urbanites [14, 15], consistent with the “healthy migrant phenomenon” shown in the international literature [16].

There is a recent concern about poor mental health status amongst young Chinese migrant workers. In a large factory of 430,000 workers, mostly migrants, in Shenzhen, southern China, there were twelve suicide attempts (all by young people aged below 30) within the five months between January and May 2010, resulting in ten deaths and two severe injuries [17]. A recent national survey by the National Bureau of Statistics of China defined “new generation” migrant workers as individuals born in 1980 or later (“post-80s”) – they were born after China’s economic reform in the late 1970s and showed a very different profile compared with older migrants, such as higher education level and being keener to become urban residents [18]. The survey also showed that there were already 85 million younger migrant workers in China in 2010. There is an urgent need to better understand psychological wellbeing in this emerging group of young migrants.

### Theories of migration and mental health

In a review of the complex relationship between migration and mental health, Kantor [19] argued that adjustments to new environments during the migratory process could be associated with improved or worsened mental health. Following this argument Kuo [20] identified four theoretical formulations that may have an impact on migrants’ mental health during the migratory process – social isolation, goal-striving stress, cultural shock, and cultural change. The latter two theoretical notions (cultural shock and cultural change) are less relevant when studying migrants who move *within* the same country and culture, as in the case of Chinese migrant workers. The theory of social isolation postulates that migrants may experience reduced social support and thus poor mental health as a result of separation from their original social networks. In contrast the theory of goal-striving stress emphasises the balance between a migrant’s aspiration and actual achievement; relatively low expectation and high subjective achievement may lead to increased satisfaction and improved mental health, whilst high expectation and low subjective achievement may lead to low satisfaction and poor mental health.

Based on this theoretical framework, we hypothesised that Chinese migrants would show better mental health compared to their urban counterparts, as the improved economic status would contribute to satisfaction and thus enhanced psychological wellbeing. These Chinese migrant workers have also been shown that they often keep a close tie with hometown communities [21] and maintain a good social network with other migrants from the same hometown [22]. We also hypothesised that younger Chinese migrant workers would have poorer mental health than their older counterparts, as they appear to be more mobile and have unstable and weaker social network [23]. Younger Chinese migrants also have higher expectation towards their lives in cities [18], and this may lead to a sense of loss and poor mental health if such expectation is not fulfilled.

The aim of this study is to investigate the mental health condition of migrant workers in Guangzhou, the provincial capital of the Guangdong province and the largest city in southern China. We have compared mental wellbeing in migrant workers with their urban counterparts and examined whether the pattern differs in younger migrants and older migrants.

## Method

### Sample

A cross-sectional study was conducted in Guangzhou city between May and July 2012. Guangzhou had a migrant population of approximately 7 million in 2011, accounting for around 50% of the city’s population [24]. “Migrant workers” were defined as individuals aged 16 years or above who have been living in Guangzhou for at least three months and do not hold a local *hukou*, i.e. with a registered residence *outside* Guangzhou. “Urban workers”, i.e. the counterparts of migrant workers, were people who have been living in Guangzhou for at least three months and do hold a local *hukou.*

We used a “quota” sampling scheme based on three occupational clusters to ensure that the sample was representative of the working population in Guangzhou; we did not intend to recruit a representative sample of the general population in Guangzhou as the focus of this study was on migrant workers. In Guangzhou, the proportions of workers employed in the manufacturing/construction, service, and business sectors were approximately 50%, 25%, and 25%, respectively [25], and we recruited participants working in the three clusters according to this distribution. As we aimed to recruit comparable groups of migrant and urban workers, the urban group was recruited from the same manufacturing units or the same service/business sectors as migrant group.

Recruitment was carried out in four of the 12 districts of the city, randomly selected to represent the inner city (Yuexiu and Tianhe), where the service and business sectors are concentrated, and suburban areas (Baiyun and Huadu) where the factories are mainly based. Migrants living in the four districts accounted for 49% of whole migrant population in Guangzhou [24]. Participants working in the service (e.g. restaurants, hotels) or business (e.g. department stores, supermarkets) sectors were recruited at the health check-up services supervised by the Guangzhou Center for Disease Control and Prevention. All workers in Guangzhou need to receive regular physical health examination, and this is a requirement for workers to obtain the working permit in the city. For subjects working in factories we first identified work units which employed both migrant and urban workers in the Baiyun and Huadu districts, and then randomly selected eight out of the 14 qualified units in Baiyun and four out of the six qualified units in Huadu. All the urban workers present on the day in each selected work unit were invited to participate in the study; the same number of migrant workers was then randomly selected from all migrant workers present on the day in the same unit. The total number of participants from one single work unit was limited to a maximum of 100. The work units included in the study represented a range of major occupations amongst migrant workers in Guangzhou: printing, cleaning product manufacturing, eyeglasses manufacturing, cement making, tyre manufacturing, shoe making, furniture manufacturing, and garment industry.

Written consent was obtained from all participants after detailed explanation of this study. The study was approved by the Ethics Committee of the Guangzhou Psychiatric Hospital, Guangzhou, China.

### Measurements

A questionnaire was used to collect information on socio-demographic characteristics, working conditions, and income. Income satisfaction was measured using a question with five possible responses – very satisfied, satisfied, average, unsatisfied, and very unsatisfied. The use of social support resources was assessed using three questions taken from the Chinese Social Support Rating Scale [26]: “Did you talk to someone for support when you felt distressed?”, “Did you seek help when you felt distressed?”, and “Are you a member of organisations such as religious group, political party, labour union, student union, etc.?”. Each question was measured on a 4-point scale with a sum score ranging between 0-12. We also collected information on whether the participants had private insurance or were covered by social security. Amongst migrant workers, we asked about motivations of migration to investigate if there was any difference in the goals and expectations between the younger and older groups. We focused on the four reasons of migration most frequently cited by participants – “wanted to earn money”, “wanted to learn skills”, “wanted to live in cities”, and “being forced by family”.

Mental wellbeing was measured using the World Health Organization Five-item Well-Being Index Scale (WHO-5) and the 5-item Mental Health subscale from the 36 Item Short Form Health Survey (SF-36). The WHO-5 includes the following five questions measuring wellbeing: “I have felt cheerful and in good spirits”, “I have felt calm and relaxed”, “I have felt active and vigorous”, “I woke up feeling fresh and rested”, and “My daily life has been filled with things that interest me” [27]. The Chinese version of the questionnaire was obtained from the WHO-5 official website (http://www.who-5.org/). The participants were asked to rate their statuses from never (score 0) to all the time (score 6) over the last two weeks, and the sum score could range between 0 to 25, with a total score below 13 suggesting a risk of poor mental health [27]. The WHO-5 has been shown to be a wellbeing scale with good reliability and validity and a sensitive screening test for depression [28] and has been used in Chinese populations [29].

The SF-36 Health Survey questionnaire has been validated in Chinese populations [30, 31]; its Mental Health (MH) subscale includes the following five questions concerning mental status: “Have you been a very nervous person?”, “Have you felt so down in the dumps that nothing could cheer you up?”, “Have you felt calm and peaceful?”, “Have you felt downhearted and blue?”, and “Have you been a happy person?”. Participants gave responses ranging from never to all the time based on the last four weeks. Responses were scored on a 6-point scale; those to the three negatively stated questions were reverse scored so that a higher score indicated better health. The sum of raw scores of the five questions was transformed to a 0-100 scale [32]. We included both the WHO-5 and SF-36 MH in the study in order to comprehensively measure mental wellbeing as prior research indicates that the two scales capture slightly different aspects of mental health [33]; for example, WHO-5 questions focus on positive affect, whilst the SF-36 MH scale includes questions about negative affect.

All the questionnaires and scales were completed by the participants on an anonymous basis. A research assistant was available to answer participants’ questions; if the participants were illiterate or had difficulty in reading, the assistant would read out the questions for the participants. The assistant would also check with the participants for illogical responses or missing values.

### Statistical analysis

We first investigated the differences between migrant and urban workers and then between younger and older migrants. Socio-demographic characteristics were compared between groups using Pearson’s χ^2^ (categorical variables) or t test (continuous variables). Linear regression models were used to investigate the difference in mental wellbeing measurements between groups and the effect of controlling for a range of potential confounders, including sex, educational qualification, marital status, job type, working hours and days, income, income satisfaction, insurance coverage, and social support. We examined whether the pattern of the migrant versus urbanite difference varied based on age cohort by fitting an interaction term between age (<= 32 years versus >32 years) and group (migrants versus urbanites) in the regression models. The cut-off point of age was decided according to the official definition of the “new generation migrant workers”, i.e. migrant workers who were born in 1980 or later, used by the National Bureau of Statistics of China in the 2010 national survey [18]. For simplicity we referred to workers aged < = 32 years at the time of the survey as “younger workers” and those aged > 32 years as “older workers”. As the primary focus of the study was on migrant workers we further investigated factors that were associated with poor mental health, indicated by a WHO-5 sum score below 13 [27], in this group using logistic regression models. Potential factors investigated included age, sex, education, marital status, factory job, working hours, working days, income, income satisfaction, insurance / welfare coverage, social support, length of stay in Guangzhou, and reasons of migration. All the analyses were conducted using Stata version 12 (StataCorp, College Station, TX, 2011).

## Results

### Migrant workers versus urban workers

A total 1856 eligible subject were invited to participate in the study and, after excluding those who refused to participate or returned questionnaires with multiple missing values, 1728 (93.1%) were eligible for analysis, including 914 migrant workers and 814 urban workers (Table 1). The mean age of migrant workers was 30 (standard deviation [SD] = 9; range 16-56) years, compared to 36 (SD = 10; range 17-60) years in urban workers; 64% of migrant workers could be classified as younger or “new generation” migrants. Migrant workers were more likely to be male and single, and had lower educational level than their urban counterparts (Table 1). The migrant group worked longer hours per day and more days per week than urbanites, whilst they earned a similar level of salary but with greater satisfaction about their income – 22% of migrants were unsatisfied or very unsatisfied with their income level, compared to 38% in urban workers. Migrant workers were less likely to have private insurance or be covered by social security than urban workers (75% versus 93%). The level of use of social support resources was lower in migrant workers than urban workers.

**Table 1:** **Socio-demographic characteristics of the participants**

Variable	Migrant workers		Urban workers		χ ^2^	df	p	Migrant workers aged < = 32 y (“new generation”)		Migrant workers aged > 32 y (“old generation”)		χ ^2^	df	p
	(N = 914)		(N = 814)					(N = 582)		(N = 332)				
	n	(%)	n	(%)				n	(%)	n	(%)			
Sex					3.2	1	0.075					0.4	1	0.52
Male	467	(51.1)	381	(46.8)				302	(51.9)	165	(49.7)			
Female	447	(48.9)	433	(53.2)				280	(48.1)	167	(50.3)			
Age (mean, SD)*	29.8	(9.1)	35.8	(10.0)	13.2	1655	<0.001							
Age group					114.0	1	<0.001							
< = 32 years (“new generation”)	582	(63.7)	309	(38.0)										
> 32 years	332	(36.3)	505	(62.0)										
Education					216.1	2	<0.001					69.6	2	<0.001
Junior high school or below	469	(51.3)	163	(20.0)				240	(41.2)	229	(69.0)			
Senior high school	341	(37.3)	386	(47.4)				253	(43.5)	88	(26.5)			
College or above	104	(11.4)	265	(32.6)				89	(15.3)	15	(4.5)			
Marital status					58.2	2	<0.001					344.2	2	<0.001
Single	421	(46.1)	230	(28.3)				402	(69.1)	19	(5.7)			
Married / cohabited	476	(52.1)	565	(69.4)				177	(30.4)	299	(90.1)			
Other	17	(1.9)	19	(2.3)				3	(0.5)	14	(4.2)			
Job					0.2	1	0.68					61.5	1	<0.001
Manufacturing	446	(48.8)	389	(47.8)				227	(39.0)	219	(66.0)			
Non-manufacturing	468	(51.2)	425	(52.2)				355	(61.0)	113	(34.0)			
Working hours per day					43.1	2	<0.001					3.5	2	0.18
< = 8	550	(60.2)	607	(74.6)				337	(57.9)	213	(64.2)			
9-11	298	(32.6)	181	(22.2)				200	(34.4)	98	(29.5)			
> = 12	66	(7.2)	26	(3.2)				45	(7.7)	21	(6.3)			
Working days per week					47.9	2	<0.001					5.3	2	0.07
< = 5	155	(17.0)	210	(25.8)				98	(16.8)	57	(17.2)			
6	498	(54.5)	475	(58.4)				332	(57.0)	166	(50.0)			
7	261	(28.6)	129	(15.8)				152	(26.1)	109	(32.8)			
Monthly income (RMB)					4.7	3	0.19					14.8	3	0.002
< 1000	52	(5.7)	41	(5.0)				23	(4.0)	29	(8.7)			
1000-3000	706	(77.2)	624	(76.7)				447	(76.8)	259	(78.0)			
3000-5000	128	(14.0)	108	(13.3)				95	(16.3)	33	(9.9)			
> = 5000	28	(3.1)	41	(5.0)				17	(2.9)	11	(3.3)			
Income satisfaction					56.8	4	<0.001					9.3	4	0.055
Very satisfied	18	(2.0)	14	(1.7)				8	(1.4)	10	(3.0)			
Satisfied	125	(13.7)	95	(11.7)				69	(11.9)	56	(16.9)			
Average	566	(61.9)	396	(48.6)				373	(64.1)	193	(58.1)			
Unsatisfied	176	(19.3)	236	(29.0)				116	(19.9)	60	(18.1)			
Very unsatisfied	29	(3.2)	73	(9.0)				16	(2.7)	13	(3.9)			
Insurance coverage**					100.4	1	<0.001					8.3	1	0.004
Yes	688	(75.3)	758	(93.1)				420	(72.2)	268	(80.7)			
None	226	(24.7)	56	(6.9)				162	(27.8)	64	(19.3)			
SSRS use of support score (mean, SD)*	7.7	(2.0)	8.0	(1.9)	3.2	1726	0.001	7.8	(1.9)	7.5	(2.0)	2.0	912	0.04
Household registration (*hukou*)												0.0	1	0.94
Urban								90	(15.5)	52	(15.7)			
Rural								492	(84.5)	280	(84.3)			
Length of stay in Guangzhou												20.3	1	<0.001
1 year or more								418	(71.8)	282	(84.9)			
Less than 1 year								164	(28.2)	50	(15.1)			
Reasons of moving to Guangzhou														
Wanted to earn more money								301	(51.7)	276	(83.1)	89.6	1	<0.001
Wanted to learn skills								299	(51.4)	94	(28.3)	45.9	1	<0.001
Wanted to live in cities								57	(9.8)	21	(6.3)	3.3	1	0.07
Forced by family								32	(5.5)	34	(10.2)	7.1	1	0.008

SD = Standard deviation. RMB = Renminbi (1 RMB ~ = 0.16 USD). SSRS = Social Support Rating Scale.

*t test used to examine group differences.

**Including private insurance and social security.

**Figure 1:**
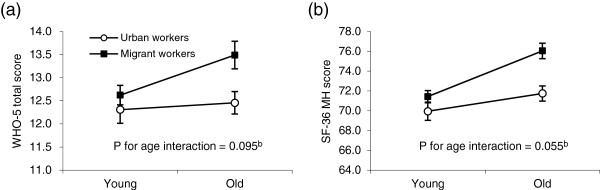
**Mean WHO-5 scores (a) and SF-36 MH scores (b) by age group, migrant workers versus urban workers** ^**a**^ **.** ^**a**^ Vertical bars indicating mean +/- standard error. ^**b**^ Adjusted for sex.

Migrant workers showed higher scores than their urban counterparts in both WHO-5 sum score (12.94 versus 12.40; difference = 0.54, 95% Confidence Interval [CI] 0.04-1.04; effect size = 0.10) and SF-36 MH scale (73.09 versus 71.06; difference = 2.04, 95% CI 0.54-3.54; effect size = 0.12) (unadjusted models in Table 2). There was some statistical evidence for the age modification effect (p for interaction = 0.095 for WHO-5 and 0.055 for SF-36 MH); better mental wellbeing in migrants than urbanites was mainly seen in the older group compared to the younger group – for example, the difference in WHO-5 sum score was 1.03 (95% CI 0.28-1.79) in the older group, compared to 0.31 (95% CI -0.39 to 1.02) in the younger group. Figure 1 shows that older migrants had the highest mental health scores, whilst younger migrants, younger urbanites, and older urbanites showed similar levels of mental wellbeing. However, even in the older group the advantage of mental wellbeing in migrant workers versus urban workers was only small according to the effect sizes (0.19 for WHO-5 and 0.25 for SF-36 MH).

**Table 2:** **Linear regression modelling analysis of WHO-5 and SF-36 MH scores, migrant workers (N = 914) versus urban workers (N = 814)**

	Unadjusted model			Adjusted model ^a^		
	β	(95% CI) ^b^	p	β	(95% CI) ^b^	p
All age groups combined						
WHO-5	0.54	(0.04, 1.04)	0.03	0.73	(0.18, 1.29)	0.01
SF-36 MH	2.04	(0.54, 3.54)	0.008	2.90	(1.22, 4.57)	0.001
Younger group (aged <= 32 years)						
WHO-5	0.31	(-0.39, 1.02)	0.38	0.72	(-0.04, 1.47)	0.06
SF-36 MH	1.47	(-0.65, 3.60)	0.17	2.57	(0.26, 4.88)	0.03
Older group (aged > 32 years)						
WHO-5	1.03	(0.28, 1.79)	0.007	0.80	(-0.05, 1.65)	0.06
SF-36 MH	4.30	(2.06, 6.54)	<0.001	3.91	(1.35, 6.47)	0.003

^a^Adjusted for sex, educational qualification, marital status, job type, working hours and days, income, income satisfaction, insurance coverage, and SSRS support score.

^b^β indicates the mean difference in scores between migrant and urban workers, with a positive value indicating higher scores or better mental health in migrant workers than urban workers and a negative value indicating the reverse.

Better mental health in migrant workers remained and even became more marked after adjusting for a range of potential confounders (adjusted models in Table 2). However, the patterns were different in the younger and older groups. Younger migrants and urbanites showed similar levels of mental wellbeing in the unadjusted models, but there was some evidence for better mental health in migrants than in urbanites in the fully adjusted model. In contrast, the advantage in older migrants versus older urbanites attenuated somewhat in the adjusted models; when we examined the effect of controlling for each of the potential confounders in separate models, the attenuation was mainly due to the controlling of income satisfaction, suggesting that part of the advantage could be attributable to greater income satisfaction in migrant workers.

### Younger migrants versus older migrants

Table 1 shows that, compared to older migrants, younger migrants had higher educational level and were more likely to be single and work in the non-manufacturing sectors. Younger and older migrants did not differ in their working hours and days. Younger migrants were more likely to be earning 3000 Renminbi (RMB) or more than older migrants (19% versus 13%), whilst they were less likely to feel satisfied or very satisfied about their income level (13% versus 20%). Compared to older migrants, younger migrants were less likely to have insurance coverage or have lived in Guangzhou for more than one year, but they were more likely to use social support resources. Young migrants were less likely to report “wanted to earn more money” (52% versus 83%) or “being forced by family” (6% versus 10%) as the motivations of migration but more likely to say that they wanted to learn skills (51% versus 28%).

Older migrants had higher mental wellbeing scores than younger migrants (WHO-5: 13.49 versus 12.62, difference = 0.87, 95% CI 0.16-1.57; SF-36 MH: 76.03 versus 71.42, difference = 4.62, 95% CI 2.64-6.60) (Table 3). The mental health advantage attenuated only to a small extent after controlling for a range of potential confounders, mainly due to the influence of marital status; this suggests that in a small part, the difference resulted from more of the older migrants being married, which was associated with better mental health. Controlling for other factors such as income satisfaction or social support use did not change the difference in younger and older groups substantially.

**Table 3:** **Linear regression modelling analysis of WHO-5 and SF-36 mental health (MH) scores, younger migrant workers versus older migrant workers (N = 914)**

	Unadjusted model			Adjusted model ^a^		
	β	(95% CI) ^b^	p	β	(95% CI) ^b^	p
WHO-5	0.87	(0.16, 1.57)	0.02	0.79	(-0.08, 1.65)	0.07
SF-36 MH	4.62	(2.64, 6.60)	<0.001	3.31	(0.80, 5.83)	0.010

^a^ Adjusted for sex, educational qualification, marital status, job, working hours and days, income, income satisfaction, insurance coverage, SSRS support score, length of stay in Guangzhou, and reasons of migration.

^b^ β indicates the mean difference in scores between younger and older migrants, with a positive value indicating higher scores or better mental health in older migrants than younger migrants and a negative value indicating the reverse.

### Factors associated with mental health

Amongst migrant workers, factors that were independently associated with poor mental health included being male, longer working hours, and being unsatisfied/very unsatisfied with income, whilst older age, factory job, high income, and increased use of social support resources were associated with reduced risk of poor mental health (Table 4).

**Table 4:** **Logistic regression modelling analysis of potential risk factors of poor mental health (WHO-5 score <13) in migrant workers (N = 914)**

Variable	Unadjusted odds ratio		p	Adjusted odds ratio		p
	(95% CI)			(95% CI) ^a^		
Age (per 10-year increase)	0.81	(0.70, 0.93)	0.003	0.67	(0.54, 0.83)	<0.001
Males	1.58	(1.21, 2.05)	0.001	1.57	(1.18, 2.09)	0.002
Lower education level (junior high school or lower)	1.25	(0.97, 1.63)	0.09	1.31	(0.96, 1.79)	0.09
Being married	0.86	(0.66, 1.12)	0.26	1.43	(0.97, 2.10)	0.07
Factory job	0.93	(0.71, 1.20)	0.57	0.73	(0.54, 0.99)	0.05
Longer working hours (> = 9 per day)	1.51	(1.15, 1.97)	0.003	1.43	(1.06, 1.92)	0.02
More working days (> = 6 per week)	1.29	(0.91, 1.83)	0.15	1.04	(0.71, 1.53)	0.85
Monthly income > = 5000 RMB	0.13	(0.04, 0.43)	<0.001	0.17	(0.05, 0.57)	0.004
Unsatisfied/very unsatisfied with income	2.00	(1.46, 2.75)	<0.001	2.13	(1.52, 2.98)	<0.001
No insurances/welfare coverage	1.22	(0.90, 1.65)	0.20	1.09	(0.79, 1.51)	0.59
SSRS use of support score (per one point increase)	0.85	(0.79, 0.91)	<0.001	0.87	(0.81, 0.94)	<0.001
Length of stay < 1 yr in Guangzhou	1.31	(0.97, 1.78)	0.08	1.21	(0.87, 1.71)	0.26
Reason of migration: wanted to earn more money	1.18	(0.90, 1.55)	0.22	1.36	(0.93, 1.98)	0.11
Reason of migration: wanted to learn skills	0.82	(0.63, 1.06)	0.13	0.98	(0.69, 1.40)	0.92
Reason of migration: wanted to live in cities	1.01	(0.64, 1.61)	0.96	1.12	(0.65, 1.91)	0.69
Reason of migration: forced by family	1.38	(0.83, 2.28)	0.21	1.23	(0.70, 2.17)	0.47

RMB = Renminbi (1 RMB ~ = 0.16 USD). SSRS = Social Support Rating Scale.

^a^Adjusted for all other variables in the table.

## Discussion

### Main findings

This study showed a small but significant advantage of mental wellbeing in migrant workers versus their urban counterparts in Guangzhou, China, whilst such advantage was found mainly in older workers compared to the younger group. Part of migrants’ advantage in mental health could be attributable to greater income satisfaction. Older migrants showed better mental health than younger migrants, and a small part of the difference was related to a higher proportion of older migrants being married. Factors that were independently associated with increased risk of poor mental health in migrants included being male, longer working hours, and income dissatisfaction, whilst older age, factory job, high income, and increased use of social support resources were associated with reduced risk.

### Migrant workers versus urban workers

The finding of better mental wellbeing in migrant workers than urban counterparts is in keeping with that from a recent study in Hangzhou [13], another economically emerging city in eastern China. In contrast, studies in Shenzhen [11] and Beijing [12] found a higher level of psychiatric symptoms in migrants than urbanites; another study in Beijing showed similar level of psychological distress in the two groups [14]. One possible reason for the different finding is methodological. Our study used scales designed to measure wellbeing, whilst some other studies [11, 12] used scales such as the Symptom Checklist-90, which is a screening test for mental symptoms or distress but not a wellbeing scale, and wellbeing is not equivalent to the absence of symptoms or distress [28]. Second, the mental health conditions of migrants may differ between Chinese cities with varying levels of economic development and income. Guangzhou had the highest average salaries amongst all cities of China [34], and relatively high income level may contribute to the advantage of mental health status amongst migrants in this study.

There are several possible explanations for the mental health advantage in migrant workers versus urban workers. First, it could result from a selection effect or “healthy migrant phenomenon” [8, 14] whereby healthy people are more likely to migrate than less healthy people [15]. However, some studies that included a rural comparison group showed that migrants actually had poorer mental health than their rural counterparts [10, 12, 13]. Second, the improvement of migrants’ economic conditions may have an important beneficial effect on mental health. Our data provide some support for this hypothesis, showing that migrants’ higher income satisfaction accounted for some of the advantage in their mental wellbeing, given the similar income level in migrants and urbanites. This suggests that income satisfaction, which depends on whether reality could match migrants’ expectations, is more important than the actual income level. Findings from studies of internal migration in Brazil [35] and Thailand [36] also suggested that the better economic opportunity due to migration may have positive impact on mental health. Third, the support network of migrants may contribute to good mental health status. Previous studies indicated that the good relationship with co-workers [13] and high level of contact with family, relatives and friends in hometown [21] were associated with better mental health amongst Chinese migrant workers, suggesting that both local network and the tie with hometown are important sources of social support for migrants.

### Younger migrants versus older migrants

The advantage of mental health in migrant workers compared to urban workers was found mainly in the older group in our study, whilst younger migrants had lower level of mental wellbeing than their older counterparts. This is in keeping with the recent concern about poor mental health in young Chinese migrants [17, 37]. There is no prior systematic investigation of mental health amongst “new generation” or younger Chinese migrant workers in comparison to older migrants [38]. A recent study of Mexican migrants in the US showed that the increased risk of depression and anxiety disorders was restricted to the younger group aged 18-35 years [39]. Another study of migrants in Australia showed that the level of mental health scores increased with age [40].

Several factors may have contributed to the lower level of mental wellbeing in younger Chinese migrants than older migrants. First, compared to older migrants, younger migrants may have lower level of social support and weaker social network. Our data showed that a small part of the disadvantage in mental health in younger versus older migrants was accounted for by a lower proportion of younger migrants being married, and marriage was shown to be a protective factor of poor mental health [13]. Second, the discrepancy between premigratory expectation and postmigratory reality may lead to a sense of loss and negative impact on mental health amongst young migrants [41]. We found that, compared to older migrants, younger migrants were less likely to report “traditional” motivations of migration such as simply wanting to make money, whilst they were more eager to learn skills, showing their interest in personal development. Despite rising expectation, many “new generation” migrants struggled with the barriers that hindered their integration in cities, such as low income and a lack of access to subsidised housing [18], partly due to the systematic discrimination resulting from the *hukou* system [38]. Therefore, “new generation” migrants may be more likely to become vulnerable to poor mental health than their older counterparts when they could not fulfil their expectation of enjoying better life quality as urban residents [38]. Third, a selection bias may contribute to the finding. Older migrants with poor mental health may already return to hometown, whilst younger migrants with poor mental health may continue to stay in the city because they have a stronger desire to become urban residents and feel less connected to rural homes [38].

### Factors associated with poor mental health in migrants

Our findings of factors related to migrants’ psychological wellbeing were generally consistent with those from previous studies; these included age [13], income [10, 11, 13], social support [13, 21, 42], and the length of working hours [43]. Previous studies also showed that Chinese migrants’ mental health was associated with marital status or marital problems [11, 13, 44], interpersonal problems [42, 44], personality traits [11], living conditions [11, 13], financial problems [42, 44], stigma or discrimination [10, 41, 45], and expectation-reality discrepancy [41]. Overall these findings highlight the role on mental health status of working and living conditions, economic status, social support, experiences of being stigmatised or discriminated, and unrealised expectations amongst Chinese migrant workers.

### Implications

Our findings have implications for social and health care policies of mental health promotion amongst Chinese migrant workers. Efforts to maintain and promote mental wellbeing amongst migrants may be usefully targeted on the younger group, addressing their specific needs such as strengthening the social support network and providing opportunities for personal development. It may be necessary to enhance migrants’ access to professional mental health care services, or for practitioners working within the existing services to actively reach out to the migrant population. A recent study showed high levels of stigma amongst community mental health staff in China [46], and anti-stigma programmes should be established amongst health staff. Many factors that are associated with poor mental health amongst Chinese migrants are related to the *hukou* system [4–6]. While some cities are already experimenting with rural-urban harmonization of the *hukou* system, recent research indicates continuous systematic stigmatisation of the migrant population [47]. The reform of the *hukou* system may substantially improve the living and working conditions as well as opportunities of migrant workers and impact positively on their mental health.

### Strengths and limitations

This is the first study showing evidence for a modification effect of age on mental wellbeing in Chinese migrant workers versus urban workers, based on a sample with a wide spectrum of occupations representative of migrant workers. There are several limitations for this study. First, the study was conducted in a relatively affluent large city in southern China, where the migrant workers may have higher satisfaction about their income and working conditions, and findings may not be generalisable to other Chinese cities. Second, quota sampling may introduce a number of biases. However it provides an efficient way to recruit a sample that includes the main occupational sectors where migrant workers are employed. Finally, the cross-sectional design was unable to study changes in mental wellbeing over the migratory process. One previous study suggests that Chinese migrants have high level of psychological distress particularly in the early stage of migration [14]. Possible adaptation over the migratory and urbanising process may play a role on migrants’ mental health, and this issue could be better studied using a longitudinal design.

## Conclusions

In this study we found a small but significant advantage in mental health in migrant workers compared to their urban counterparts in Guangzhou, China. Part of this advantage was due to greater income satisfaction in migrant workers. However, such advantage was not seen in younger migrants compared to younger urbanites. Amongst migrants, a range of socio-demographic factors, working condition, income and income satisfaction, and social support were associated with mental health condition. Efforts to promote mental health amongst migrant workers may be usefully targeted on younger migrants and include measures aimed to improve working conditions, strengthen the social support network, and address age-specific needs.

## Abbreviations

WHO-5: World Health Organization Five-item Well-Being Index Scale
SF-36: The 36 Item Short Form Health Survey
SF-36 MH: Mental Health subscale from SF-36
CI: Confidence interval.
